# Effect of bovine colostrum feeding in comparison with milk replacer and natural feeding on the immune responses and colonisation of enterotoxigenic *Escherichia coli* in the intestinal tissue of piglets

**DOI:** 10.1017/S0007114514003201

**Published:** 2015-03-06

**Authors:** Sugiharto Sugiharto, Ann-Sofie Riis Poulsen, Nuria Canibe, Charlotte Lauridsen

**Affiliations:** 1 Department of Animal Science, Faculty of Science and Technology, University of Aarhus, 8830Tjele, Denmark; 2 Faculty of Animal and Agricultural Sciences, Diponegoro University, Semarang, Central Java50275, Indonesia

**Keywords:** Bovine colostrum, Diarrhoea, Enterotoxigenic *Escherichia coli*, Intestinal immunity, Weak piglets

## Abstract

The present study investigated the effect of feeding bovine colostrum (BC) to piglets in comparison with feeding a milk replacer (MR) and conventional rearing by the sow on the intestinal immune system and number of enterotoxigenic *Escherichia coli* (ETEC) colonising the intestinal tissue. Piglets (23-d-old) were allocated to one of the following four groups: (1) killed at the beginning of the experiment (Base); (2) separated from the sow and fed BC (BC-fed); (3) separated from the sow and fed a MR (MR-fed); (4) kept with the sow (Sow-Milk). Blood was sampled on days 1 and 8, and faecal samples were collected on days 1, 3, 5 and 8. On day 8, piglets were killed and gastrointestinal digesta and intestinal segments were collected. The frequency of diarrhoea was found to be higher (*P*≤ 0·019) in MR-fed piglets than in BC-fed and Sow-Milk piglets. Piglets from the MR-fed group had the lowest lactic acid bacteria:haemolytic *E. coli* ratio (*P*_treat_= 0·064) in the faeces. The number of *E. coli* colonising the intestinal tissue was higher (*P*< 0·001) in piglets from the MR-fed group than in those from the BC-fed and Sow-Milk groups. Piglets from the Sow-Milk group had a higher (*P*= 0·020) mucosal IgG concentration than those from the MR-fed group, but did not exhibit any difference when compared with piglets from the Base and BC-fed groups. Piglets from the BC-fed group exhibited a reduced (*P*≤ 0·037) expression level of Toll-like receptor-4 in the intestinal mucosa when compared with those from the MR-fed and Sow-Milk groups. The expression level of *IL-2* was higher (*P*≤ 0·051) in piglets from the MR-fed group than in those from the other treatment groups. In conclusion, feeding BC rather than MR to the piglets reduced the colonisation of intestine by ETEC and modulated the intestinal immune system, whereas no differences were observed in piglets fed BC and conventionally reared by the sows.

Increasing the litter size has long been a goal among pig producers, as the number of offspring born is an important economic trait. Initially, improvements made in management and nutrition were aimed at increasing the litter size, and more recently, genetic selection for litter size has been implemented^(^ ^1^ ^)^. It has been reported that an increased litter size is genetically associated with a decreased mean birth weight and an increased within-litter birth weight variation^(^ ^2^ ^)^. Therefore, the use of hyperprolific sows in the pig industry not only results in large litters, but also leads to an increase in the number of supernumerary and underprivileged piglets (underweight at birth, referred here as weak piglets)^(^ ^3^ ^)^.

Apart from its value as a complete nutrient source, milk contains a wide range of factors (e.g. antibodies and cytokines) that can modulate the development and maturation of the immune system and subsequently the immune response of piglets^(^ ^4^ ^,^ ^5^ ^)^. During lactation, lower milk supply per piglet is often observed in large litters. This particularly occurs after the maximal milk production capacity is attained (7–15 d)^(^ ^6^ ^)^. Given that weak piglets have a lower ability to compete for the udder^(^ ^7^ ^)^ and are often less vigorous in suckling^(^ ^6^ ^)^, their milk intake may be insufficient^(^ ^7^ ^)^. Hence, it is not surprising that weak piglets have a poor pre-weaning growth performance and often fail to thrive, or even die, by the time of weaning^(^ ^8^ ^)^. It has been suggested that low body weight and immunoincompetence at weaning adversely affect the growth performance and viability of piglets after weaning^(^ ^7^ ^,^ ^9^ ^,^ ^10^ ^)^. Thus, efforts to enhance the growth and survival of weak piglets after weaning should be aimed at improving not only the weaning weight, but also the health and immunity^(^ ^11^ ^)^.

Bovine colostrum (BC), which is a commercially available co-product from the dairy industry, is known to contain several growth factors as well as antimicrobial and immunomodulatory components essential for host development and defence functions^(^ ^4^ ^)^. Of the bioactive components, Ig, especially IgG, appear to be the most important immune components present in BC as they represent 70–80 % of the total protein content in the colostrum^(^ ^4^ ^)^. Bovine colostral Ig provide the host with passive immunity^(^ ^12^ ^,^ ^13^ ^)^ and, in concert with lactoferrin, lactoperoxidase and lysozyme, may act as antimicrobial agents^(^ ^4^ ^)^. BC also contains high amounts of cytokines that can modulate the development and maturation of the immune system^(^ ^4^ ^,^ ^5^ ^)^. Besides the provision of supplemental milk during suckling^(^ ^11^ ^)^, artificial rearing on milk replacers (MR)^(^ ^3^ ^)^ has been reported as a good alternative for raising low-birth-weight piglets before weaning. Taking this into consideration, rearing weak piglets artificially on BC could be expected to improve the growth performance as well as to boost the immune system before weaning. The aim of the present study was to investigate the effect of feeding a commercial BC product to piglets in comparison with feeding a common MR and conventional rearing by the sow on the intestinal immune system and colonisation of ETEC in the intestinal tissue. Our hypothesis was that BC would be a better source than MR and as good as conventional rearing by the sow in terms of optimisation of the immune defence and intestinal health of weak piglets.

## Materials and methods

### Experimental design

The present experiment was conducted according to the licence obtained from the Danish Animal Experiments Inspectorate, Ministry of Food, Agriculture and Fisheries, Danish Veterinary and Food Administration.

A total of forty piglets (8·30 (sd 1·45) kg) from four litters obtained from the herd at the Faculty of Science and Technology, Aarhus University, Foulum (Denmark), were used in the present experiment. All piglets were from sows tested homozygote carriers of the dominant gene encoding for the intestinal F18 fimbriae receptors (a DNA marker genotyping-based test was performed on DNA extracted from hair samples by van Haeringen laboratorium b.v., Wageningen, The Netherlands); thus, piglets used in the present study were susceptible to *Escherichia coli* F18. Piglets were provided neither additional milk nor creep feed from birth to weaning (separation from the sow).

Of the forty piglets, four piglets, one from each litter, were killed at the beginning of the experiment (Base) and thirty-six piglets, nine per litter, were kept alive and randomly allocated to one of the following three treatment groups: (1) separated from the sow and fed a commercial BC product (BC-fed); (2) separated from the sow and fed a commercial MR (MR-fed); (3) kept with the sow (Sow-Milk). Piglets from the BC-fed and MR-fed groups were separated from the sows at 23 d of age, transferred to the experimental stable and provided either skimmed standardised BC (European Colostrum Industry S. A.) or a commercial MR (Vitfoss) *ad libitum* for 8 d. The chemical composition of the sow milk, BC and MR is given in Table 1. Liquid BC and MR were prepared using an automated wet feeder (Mambo Automix 25; Wit-Mambo, Inc.), in which BC and MR powders were automatically mixed with warm water (45°C; approximately 150 g of powder in 1 litre of water) and given to the piglets on a regular basis. To help the piglets become familiar with the automated wet feeder and stimulate the consumption of liquid BC or MR, sow milk collected from the corresponding sow was fed to the piglets using the feeder upon arrival to the experimental stable. Piglets from the Sow-Milk group were kept with their dams in the farrowing unit until the end of the experiment without any supplementary feed or milk. To minimise the variation among the treatment groups in terms of stress caused by separation from the sow, piglets from the Sow-Milk group were transported in a similar manner to those in the other groups and returned to the sows again.

**Table 1:** Chemical composition of the bovine colostrum (BC), milk replacer (MR) and sow milk

Items	BC*	MR*	Sow milk†
DM (%)	96·1	95·0	17·9
Crude protein (% DM)	71·0	23·3	28·5
Crude fat (% DM)	2·1	13·9	36·3
Ash (% DM)	6·2	7·2	5·6
Ig (% DM)‡			
IgG	38·4	0·05	0·11
IgA	3·59	0·01	2·18
IgM	2·52	ND	0·56

ND, not detected (detection level = 78 ng/ml, equivalent to 0·00 001 %).

* Chemical analyses of BC and MR were performed by Eurofins Steins Laboratorium A/S, Odense, Denmark.

† The chemical compositions of sow milk, especially DM, protein and fat, are adopted from Lauridsen & Danielsen^(^ ^38^ ^)^, while that of ash is adopted from Aguinaga *et al.* ^(^ ^39^ ^)^.

‡ The concentrations of Ig were determined in our laboratory. Values of Ig for sow milk are the average values from four milk samples (obtained from four sows).

A total of three littermates of similar weight were housed together in 1·45 × 1·70 m^2^ pens with rubber mat and sawdust bedding. Each pen was equipped with an automated wet feeder and a water nipple giving permanent free access to feed and water to all the three piglets. Temperature in the experimental stable was maintained at 24°C throughout the experiment and the pens were equipped with a heat lamp. To reduce the impact of moving BC-fed and MR-fed piglets from the sows when compared with Sow-Milk piglets regarding microbial contamination from the environment, material (a mixture of bedding and faeces) from the floor of pens where the sows were housed was collected daily and spread in the corresponding pens where BC-fed and MR-fed piglets were housed. In this way, all piglets were continuously exposed to the microbiota of sow faecal origin.

### Sampling and data collection

The body weight of each piglet was recorded at the beginning (day 1) and at the end (day 8) of the experiment. BC and MR consumption was measured by recording the weight of the powder provided to the piglets and any unconsumed residue on a daily basis. For Ig analyses, blood samples were collected in heparinised Vacutainer tubes by puncture of the jugular vein of each piglet on days 1 and 8. Plasma was obtained after centrifugation of blood samples at 2000 ***g*** for 10 min and stored at − 20°C until analysis. Faecal samples were collected from the rectum of each piglet on days 1, 3, 5 and 8 of the experiment. Rectal temperature was recorded with a digital thermometer (Kruuse) before faecal sample collection. The consistency of faecal samples (1 = hard, dry and cloddy; 2 = firm; 3 = soft with shape; 4 = soft and liquid; 5 = watery and dark; 6 = watery and yellow; and 7 = foamy and yellow), used as an indicator of the occurrence of diarrhoea, was recorded immediately after sample collection. A faecal consistency score >3 was defined as clinical signs of diarrhoea^(^ ^14^ ^)^. The frequency of diarrhoea was calculated by counting pig days with a diarrhoea score >3. The alertness score (0 = normal and 1 = depressed or listless) of each piglet was also assessed visually throughout the experiment.

One piglet per litter (four in total) was killed using a captive bolt gun at the beginning of the experiment (day 1) and one piglet per litter and treatment (12 in total) was killed at the end of the experiment (day 8). The abdomen was incised and gall bladder (for bile collection) was obtained immediately after bleeding the animal. The gastrointestinal tract (GIT) was immediately removed and divided into five segments (stomach, proximal and distal small intestine, caecum and mid-colon), and digesta from each segment was collected for bacterial enumeration. The length of the small intestine, from the pyloric sphincter to the ileocolonic junction, was measured before digesta collection. Approximately 15 cm segments from 10 % (representing duodenum), 50 % (jejunum) and 90 % (ileum) of the length of the small intestine measured from the duodenum were removed, opened lengthways and washed with ice-cold PBS. Mucosa was collected from these segments by gently scraping with a glass microscope slide and immediately stored at − 20°C until Ig analyses. For gene expression analyses, mucosa (approximately 100 mg) collected from the same sites of the small intestine (jejunum and ileum) was preserved in the RNAlater (1 ml; Sigma-Aldrich) and stored at 4°C for 1 d before storage at − 20°C. Intestinal segments (jejunum and ileum) were also collected from piglets killed at the end of the experiment and used in the porcine intestinal organ culture (PIOC) experiment.

### Porcine intestinal organ culture experiment

A total of twelve piglets, one per litter and treatment, were used in the PIOC experiment. Immediately after incising the abdomen and bleeding the animal, three jejunal segments and three ileal segments (15 cm each) were collected aseptically, immersed in Dulbecco's modified Eagle's medium (DMEM), kept on ice and immediately used in the PIOC experiment. *E. coli* F18 inoculum was prepared by retrieving the bacterial culture stored at − 80°C, streaking on blood agar (Oxoid, Deutschland GmbH) and culturing at 37°C for 18 h. A loopful of *E. coli* F18 colony was taken from blood agar and suspended in 4 ml of PBS. The suspension (0·1 ml) was poured onto Iso-Sensitest agar (Oxoid) and incubated at 37°C overnight. PBS (10 ml) was poured into the incubated plate, and the agar surface was gently rubbed with a sterile Drigalski spatula to remove the bacterial colonies from the agar plate. The bacterial suspension was transferred into a sterile tube and diluted 1:20 (v/v) with DMEM to obtain an inoculum of 5 × 10^8^ colony-forming units (cfu)/ml before being used for inoculating the intestinal segments of piglets. The *E. coli* F18 strain was isolated at the Danish Veterinary Institute (Frederiksberg, Copenhagen, Denmark) from the intestinal content of a pig with post-weaning diarrhoea. It was found to harbour genes for enterotoxins heat stable toxin b (STb), heat labile toxin (LT), Enteroaggregative *Escherichia coli* heat-stable enterotoxin 1 (EAST1), Shiga toxin 2e and fimbriae F18. The bacteria were found to be haemolytic when grown on blood agar (Oxoid)^(^ ^15^ ^)^.

Of the collected jejunal and ileal segments, two pieces were inoculated with *E. coli* F18 and one piece was used as a control (not inoculated). The PIOC experiment was conducted based on the model described by Naughton *et al.* ^(^ ^16^ ^)^ and modified by Sugiharto *et al.* ^(^ ^17^ ^)^. In brief, polyethylene tubing (Siltube; Eurpharm; 6 mm in diameter) was inserted into either end of the segment and tied with a suture to keep the tubing in place. The tissue was washed with 50 ml of PBS (pH 7·2) using a Fill-Master pump (Type 311; Delta Scientific Medical; flow rate of 7·7 cm/s). The other end of the segment was tied, 10 ml of DMEM alone (control) or DMEM containing *E. coli* F18 was inoculated, and the segment was sealed with a Teflon plug (5 mm in diameter). The segment was immersed in DMEM in a 300 ml infusion bottle in a shaking water-bath at 37°C, removed after 1·5 h and washed with 50 ml of PBS after removing the content. Mucosa was collected from a piece of the segment and preserved in the RNAlater for gene expression analyses. The remaining segment was weighed and homogenised (Janke-Kunkel Ultra-Turrax T25 homogeniser; Bie&Bentsen a/s) for 20 s in PBS. *E. coli* were enumerated on MacConkey agar (Merck KGaA) after aerobic incubation at 37°C overnight.

### Microbiological analyses of the faecal and digesta samples

Microbiological analyses were performed on faecal samples (collected daily from one piglet per pen) and digesta samples collected from piglets that were killed at the end of the experiment. Approximately 1 g of faeces and 10 g of digesta were suspended in a pre-reduced salt medium^(^ ^18^ ^)^ and homogenised in a laboratory paddle blender (Seward Stomachers^®^ 80 Biomaster; Lab System) for 2 min. Serial 10-fold dilutions were then prepared in the same medium and samples (0·1 ml) were plated on selective media. Haemolytic *E. coli* and coliform bacteria were enumerated on blood agar (Oxoid) and MacConkey agar (Merck), respectively, after aerobic incubation at 37°C for 1 d, whereas lactic acid bacteria (LAB) were enumerated on de Man–Rogosa–Sharpe agar (Merck) after anaerobic incubation at 37°C for 3 d. The number of *E. coli* and LAB is presented as the LAB:haemolytic *E. coli* ratio or the LAB:total coliform bacteria ratio.

### Immunoglobulin analyses

The concentrations of total IgG, IgA and IgM in the mucosa, plasma and sow milk and those of IgA and IgM in bile were measured using the pig Ig ELISA quantification kit (Bethyl Laboratories). The same Ig types were measured in the skimmed standardised BC and commercial MR using the bovine Ig ELISA quantification kit (Bethyl Laboratories). All assays were performed according to the manufacturer's instructions and run in duplicates. The intra-assay (within-plate) CV for the ELISA was 0·1–12·5 % and the inter-assay (between-plate) CV was 0·2–12·3 %. Before the assays, the mucosa samples were vortexed in PBS (1:10, w/w) for 60 s and centrifuged at 2000 ***g*** for 10 min and the supernatant was obtained and used for the determination of Ig concentrations and total protein content. The concentration of IgA in the intestinal mucosa is expressed as the relative amount of IgA to that of total protein. Total protein content in the mucosa was quantified using the Advia 1650 autoanalyser (Siemens Medical Solutions).

### Gene expression analyses

The mucosa was homogenised by transferring it into a lysis buffer (Macherey-Nagel GmbH & Co. KG) and treated with a rotor–stator homogeniser (TissueLyser LT; Qiagen GmbH) until it was uniformly homogeneous. Total RNA was isolated using the NucleoSpin RNA kits (Macherey-Nagel) according to the manufacturer's protocols. The quality and quantity of RNA were assessed using agarose gel electrophoresis and a spectrophotometer (NanoDrop ND-1000; Saveen Werner), respectively. All samples were adjusted to 200 ng/μl and converted into complementary DNA using the High Capacity cDNA Reverse Transcription Kit (Invitrogen) according to the manufacturer's instructions. All PCR were run in 384-well plates by mixing 2 μl of complementary DNA with 8 μl of the mix containing the primers, the probe and 2 ×  TaqMan Master Mix (catalogue no. 4324018; Applied Biosystems) (Table 2). The reactions were run in duplicate for the target genes and triplicate for the housekeeping gene under standard amplification conditions determined for the Applied Biosystem ViiA 7 Real-Time PCR system (Life Technologies). Gene expression cycle threshold (*C*_t_) values were recorded using the Applied Biosystem ViiA 7 software. Glyceraldehyde-3-phosphate dehydrogenase (GAPDH) was used as the housekeeping gene. The *C*_t_ value for the target genes of each sample was corrected by subtracting the *C*_t_ value of the *GAPDH* gene from the *C*_t_ value of the corresponding gene (Δ*C*_t_). The samples collected from the jejunal mucosa of Base piglets were used as the reference samples, and the Δ*C*_t_ value of all the samples was subtracted from the average Δ*C*_t_ value of the reference samples (ΔΔ*C*_t_). The fold change in the expression of each target gene was calculated using the following formula: 2^− ΔΔ*C*_t_^.

**Table 2:** Sequences of primers and probes used in real-time PCR

Targets	Primer sequences	Accession no.
*GAPDH*	Forward: 5′**-**GTCGGAGTGAACGGATTTGG-3′	AF017079
	Reverse: 5′**-**CAATGTCCACTTTGCCAGAGTTAA-3′	
	Probe: 5′**-**CGCCTGGTCACCAGGGCTGCT-3′	
*IL-10*	Forward: 5′**-**GAGGAGGTGAAGAGTGCCTTTA-3′	L20001
	Reverse: 5′**-**CTCACCCATGGCTTTGTAGACA-3′	
	Probe: FAM**-**CCTCTCTTGGAGCTTGC**-**MGB	
*TNF-α*	Forward: 5′**-**AACCCTCTGGCCCAAGGA-3′	X57321
	Reverse: 5′**-**GGCGACGGGCTTATCTGA-3′	
	Probe: FAM**-**TCAGATCATCGTCTCAAAC**-**MGB	
*IL-2*	Forward: 5′**-**GATCTCTCCAGGATGCTCACATTT-3′	X56750
	Reverse: 5′**-**CTCCAGAGCTTTGAGTTCTTCTACT-3′	
	Probe: FAM**-**CCCAAGCAGGCTACAGAA**-**MGB	
*COX-2*	Forward: 5′**-**GGGACGATGAACGGCTGTT-3′	NM_214321
	Reverse: 5′**-**CACAATCTTAATCGTTTCTCCTATCAGT-3′	
	Probe: 5′**-**AGACGAGCAGGCTGA-3′	
*TLR-4*	20 × TagMan^®^ Gene Expression Assay, catalogue no. 4331182 (Applied Biosystems)	
*TGF-β1*	20 × TagMan^®^ Gene Expression Assay, catalogue no. 4331182 (Applied Biosystems)	

*GAPDH*, glyceraldehyde-3-phosphate dehydrogenase; *COX-2*, cyclo-oxygenase-2; *TLR-4*, Toll-like receptor-4; *TGF-β1*, transforming growth factor-β1.

### Calculations and statistical analyses

Data on weight gain, frequency of diarrhoea, rectal temperature, alertness score and plasma Ig concentrations were obtained from each piglet, while data on microbial counts, mucosal and biliary Ig concentrations, and gene expression levels were obtained from one piglet per pen. Bacterial population counts (cfu) were normalised using log transformation, and data on weight gain of each piglet were collected per pen before analysis. Data on bacterial counts obtained from ETEC F18-inoculated jejunal or ileal samples (PIOC experiment) were averaged per piglet before analysis.

The frequency of diarrhoea and alertness scores were analysed using the GLIMMIX procedure of SAS (SAS Institute) with treatment as a fixed effect and litter and pen as the random effects to account for the measurement being made in piglets from different litters and different pens. Data on performance and biliary Ig concentrations were analysed with a mixed model using the MIXED procedure of SAS with treatment and litter as the fixed and random effects, respectively. The remaining data that were obtained on various days (rectal temperature, LAB:*E. coli* ratio in faeces and plasma Ig concentrations) or from various segments of the gut (LAB: *E. coli* ratio in the digesta, number of *E. coli* colonising the intestine, mucosal Ig concentrations and gene expression levels) of the same piglet were analysed using the MIXED procedure with treatment and sampling day or gut/intestinal site as the fixed effects and litter as a random effect and taking into account that samples originated from the same animal. For data on rectal temperature and plasma Ig concentrations, which were obtained from piglets from different litters and pens, the random effects of litter and pen were imposed. The interaction between treatment and sampling day or gut/intestinal site was initially included in the model, but excluded when it was not statistically significant. Differences among the treatment groups were tested using the *post hoc* Tukey–Kramer test. Differences were considered significant when *P*≤ 0·05. Results are presented as least-squares means and standard errors.

## Results

### Growth performance and frequency of diarrhoea

During the experiment, two piglets (from the BC-fed and MR-fed groups) died and so data obtained from these piglets were not included in the analyses. Piglets that were kept with the sows, i.e. the Sow-Milk group, exhibited a higher (*P*≤ 0·029) weight gain compared with those separated from the sows and fed BC or MR (Table 3). The frequency of diarrhoea was higher (*P*≤ 0·019) in MR-fed piglets than in BC-fed and Sow-Milk piglets (Table 3). There were no significant differences with regard to the alertness of piglets throughout the experiment (data not shown). The rectal temperature did not differ significantly among the treatment groups throughout the experiment (ranged from 37 to 40°C; data not shown).

**Table 3:** Growth performance and frequency of diarrhoea in piglets in response to the treatments (Least-squares means with their standard errors)

	Treatments		
Items	BC-fed	MR-fed	Sow-Milk	se	*P*
Weight gain (g)*	2024^a^	3023^a^	6440^b^	1649	0·023
Milk intake (g)*	2981	4767	NR	1835	0·133
Pig days†	44	44	48		
Frequency of diarrhoea‡	5^a^	18^b^	3^a^		0·008

BC-fed, piglets separated from the sow and fed bovine colostrum; MR-fed, piglets separated from the sow and fed a commercial milk replacer; Sow-Milk, piglets kept with the sow; NR, not recorded.

^a,b^Values within a row with unlike superscript letters were significantly different (*P*≤ 0·05).

* Data presented are the weight gain and milk intake recorded in each pen throughout the experimental period. BC-fed, *n* 3 pens; MR-fed, *n* 3 pens; Sow-Milk, *n* 4 pens. Data collected from pens with only two piglets (due to the death of one piglet) were not included in the statistical analyses.

† Pig days = number of piglets × number of days of diarrhoea scoring. BC-fed, *n* 11 piglets; MR-fed, *n* 11 piglets; Sow-Milk, *n* 12 piglets. Diarrhoea scoring was conducted on days 1, 3, 5 and 8 of the experiment.

‡ Frequency of diarrhoea = number of pig days with diarrhoea score >3.

### Ratios of lactic acid bacteria:Escherichia coli in the faeces and gastrointestinal digesta

**Fig. 1:**
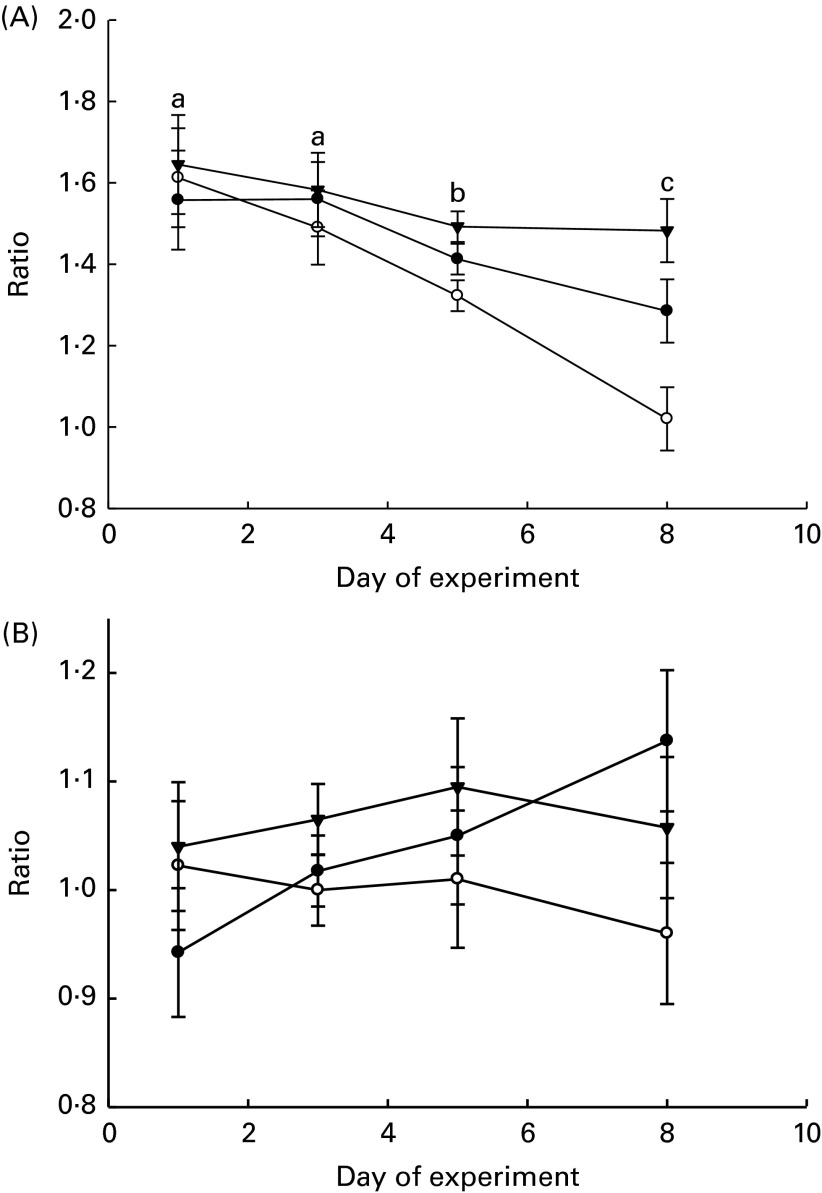
Lactic acid bacteria:haemolytic *Escherichia coli* ratio (A) and lactic acid bacteria:total coliform bacteria ratio (B) in the faeces of piglets. Values are least-squares means, with their standard errors represented by vertical bars. ^a,b,c^Mean values with unlike letters were significantly different (*P*≤ 0·05). Four piglets were analysed per treatment and day of experiment (faecal sampling). BC-fed (–●–), piglets separated from the sow and fed bovine colostrum; MR-fed (–○–), piglets separated from the sow and fed a commercial milk replacer; Sow-Milk (–▾–), piglets kept with the sow. (A) *P*_treat_= 0·064; *P*_day_= 0·029; *P*_treat × day_= 0·325. (B) *P*_treat_= 0·143; *P*_day_= 0·784; *P*_treat × day_= 0·626.

**Fig. 2:**
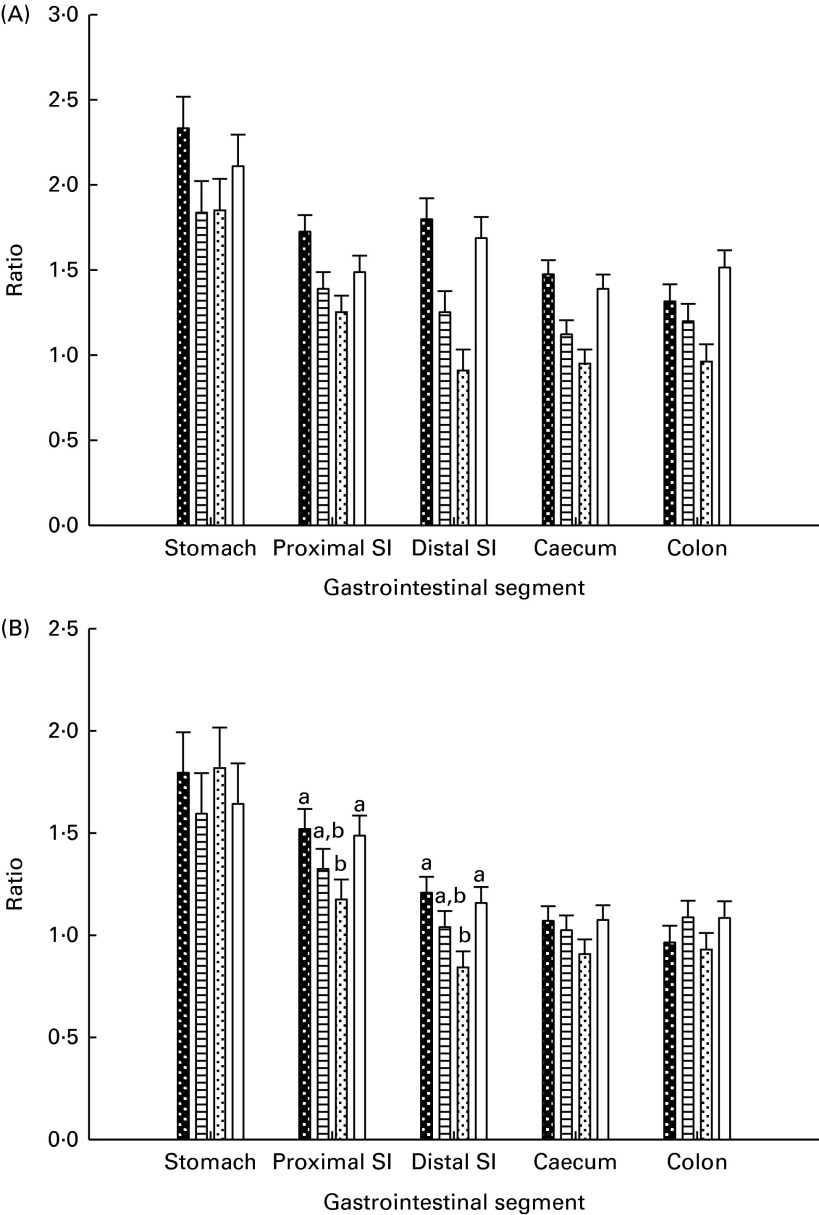
Lactic acid bacteria:haemolytic *E. coli* ratio (A) and lactic acid bacteria:total coliform bacteria ratio (B) in the gastrointestinal digesta of piglets. Values are least-squares means, with their standard errors represented by vertical bars. ^a,b^Mean values with unlike letters were significantly different (*P*≤ 0·05). Four piglets were analysed per treatment and gastrointestinal segment. Base (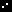), piglets killed at the beginning of the experiment; BC-fed (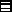), piglets separated from the sow and fed bovine colostrum; MR-fed (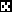), piglets separated from the sow and fed a commercial milk replacer; Sow-Milk (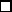), piglets kept with the sow. SI, small intestine. (A) *P*_treat_= 0·068; *P*_seg_< 0·001; *P*_treat × seg_= 0·055. (B) *P*_treat_= 0·302; *P*_seg_< 0·001; *P*_treat × seg_= 0·033.

The ratios of LAB:haemolytic *E. coli* and LAB:total coliform bacteria in the faeces and GIT digesta of piglets are shown in Figs. 1 and 2, respectively. The LAB:haemolytic *E. coli* ratio was higher (*P*_day_= 0·029) during the first 3 d of the experiment than in the remaining 5 d and tended (*P*_treat_= 0·064) to be lower in the faeces of MR-fed piglets than in that of BC-fed and Sow-Milk piglets. The Base and Sow-Milk piglets had a higher LAB:coliform bacteria ratio in the digesta collected from the small intestine when compared with MR-fed piglets (*P*≤ 0·043), but exhibited no differences when compared with BC-fed piglets. The LAB:haemolytic *E. coli* ratio in the gastrointestinal digesta of MR-fed piglets exhibited a decreasing tendency (*P*_treat_= 0·068) when compared with that in piglets from the other treatment groups.

### Number of Escherichia coli colonising the intestinal tissue

The number of *E. coli* colonising the jejunal and ileal tissue and content is summarised in Table 4. The number of *E. coli* was higher (*P*≤ 0·039) in the jejunal and ileal tissue and content in the non-inoculated intestinal samples of MR-fed piglets than in the samples of BC-fed and Sow-Milk piglets. The number of *E. coli* was higher (*P*_seg_< 0·001) in the ileal tissue and content than in the jejunal tissue and content. The number of *E. coli* colonising the intestinal tissue in the ETEC F18-inoculated jejunal and ileal samples of BC-fed piglets was lower than that in the samples of MR-fed piglets, but there were no significant differences in values (*P*_treat_= 0·320).

**Table 4:** Number of *Escherichia coli* colonising the jejunal or ileal tissue and content in the porcine intestinal organ cultures of samples collected from the treated piglets (Least-squares means with their standard errors)

	Treatments*		*P*
Items	BC-fed	MR-fed	Sow-Milk	se	Treat	Seg	Treat × seg
Non-inoculated tissue (log cfu/g)†					< 0·001	< 0·001	0·500
Jejunum	4·25^a^	5·91^b^	4·72^a^	0·46			
Ileum	6·61^a^	7·65^b^	6·09^a^	0·46			
Non-inoculated content (log cfu/ml)†					0·020	< 0·001	0·860
Jejunum	3·52^a^	5·00^b^	4·00^a^	0·71			
Ileum	6·10^a^	7·20^b^	6·10^a^	0·71			
*E. coli*-inoculated tissue (log cfu/g)‡					0·320	0·470	0·330
Jejunum	7·80	8·13	7·57	0·33			
Ileum	7·55	8·27	8·18	0·33			
*E. coli*-inoculated content (log cfu/ml)‡					0·590	0·070	0·450
Jejunum	8·92	9·03	9·13	0·10			
Ileum	8·89	8·91	8·92	0·10			

BC-fed, piglets separated from the sow and fed bovine colostrum; MR-fed, piglets separated from the sow and fed a commercial milk replacer; Sow-Milk, piglets kept with the sow; Treat, treatment; Seg, segment of the small intestine; cfu, colony-forming units.

^a,b^Values within a row with unlike superscript letters were significantly different (*P*≤ 0·05).

* Four piglets were analysed per treatment (of each small-intestinal site, one segment was not inoculated and two segments were inoculated with *Escherichia coli* F18).

† Intestinal segments (collected from the treated piglets) were not inoculated with *E. coli* F18 during the porcine intestinal organ culture (PIOC) experiment.

‡ Intestinal segments (collected from the treated piglets) were inoculated with *E. coli* F18 (8·96 (se 0·29) log colony-forming units/ml for each segment) during the PIOC experiment.

### Ig concentrations in the intestinal mucosa, bile and plasma

The concentrations of total Ig in the intestinal mucosa and bile of piglets are given in Table 5. Piglets from the Sow-Milk group had a higher (*P*= 0·020) concentration of IgG in the mucosa when compared with piglets from the MR-fed group, but did not exhibit any difference when compared with piglets from the Base and BC-fed groups. The concentration of mucosal IgA was found to be influenced by the interaction between treatment and intestinal segment (*P*_treat × seg_= 0·087), as evidenced by the higher concentrations of IgA in the jejunum and ileum of Base piglets than in those of piglets from the other treatment groups. Piglets from the MR-fed group had a lower (*P*= 0·051) concentration of total IgM in the mucosa when compared with those from the Base group, but did not exhibit any difference when compared with piglets from the BC-fed and Sow-Milk groups. Regardless of treatment, the concentration of total IgM was higher (*P*≤ 0·045) in the ileum than in the duodenum or jejunum, while that of IgA was higher (*P*= 0·034) in the ileum than in the duodenum, but did not differ from that in the jejunum. No significant differences were observed with regard to the concentrations of IgA (*P*= 0·236) and IgM (*P*= 0·062) in the bile of piglets; however, Base piglets tended (*P*≤ 0·089) to have a lower concentration of IgM when compared with piglets from the other treatment groups.

**Table 5:** Concentrations of total Ig in the intestinal mucosa and bile of piglets in response to the treatments (Least-squares means with their standard errors)

	Treatments*		*P*
Items	Base	BC-fed	MR-fed	Sow-Milk	se	Treat	Seg	Treat × seg
IgG (mg/g)						0·030	0·963	0·268
Duodenum	0·66^a,b^	0·85^a,b^	0·62^a^	0·80^b^	0·27			
Jejunum	0·93^a,b^	0·50^a,b^	0·86^a^	0·74^b^	0·24			
Ileum	0·60^a,b^	0·68^a,b^	0·36^a^	1·21^b^	0·13			
IgA (mg/g)						0·160	0·031	0·087
Duodenum	1·88	1·85	1·92	3·15	0·47			
Jejunum	13·35	1·43	1·17	2·35	3·10			
Ileum	11·48	3·15	1·80	4·80	2·11			
IgM (mg/g)						0·051	0·001	0·110
Duodenum	1·73^a^	2·76^a,b^	4·36^b^	5·41^a,b^	1·22			
Jejunum	2·23^a^	2·34^a,b^	2·94^b^	3·34^a,b^	0·72			
Ileum	8·70^a^	5·27^a,b^	3·98^b^	5·41^a,b^	0·94			
Biliary IgA (mg/l)	2·9	13·0	22·5	10·8	6·9	0·236		
Biliary IgM (mg/l)	1·4	4·9	6·8	4·6	1·5	0·062		

Base, Piglets killed at the beginning of the experiment; BC-fed, piglets separated from the sow and fed bovine colostrum; MR-fed, piglets separated from the sow and fed a commercial milk replacer; Sow-Milk, piglets kept with the sow; Treat, treatment; Seg, segment of the small intestine.

^a,b^Values within a row with unlike superscript letters were significantly different (*P*≤ 0·05).

* Four piglets were analysed per treatment and small-intestinal site.

There were no significant differences in the plasma concentrations of Ig among the treatment groups. Irrespective of the treatment, the concentration of IgG was lower (*P*_day_< 0·001) on day 8 than on day 1 (Table 6).

**Table 6:** Concentrations of total Ig in the plasma of piglets in response to the treatments (Least-squares means with their standard errors)

	Treatments		*P*
Items	BC-fed (*n* 11)	MR-fed (*n* 11)	Sow-Milk (*n* 12)	se	Treat	Day	Treat × day
IgG (mg/l)					0·606	< 0·001	0·944
Day 1	6120	5470	6020	640			
Day 8	3960	3440	3740	480			
IgA (mg/l)					0·719	0·173	0·877
Day 1	200	200	220	30			
Day 8	170	170	210	40			
IgM (mg/l)					0·447	0·194	0·892
Day 1	690	710	890	150			
Day 8	880	840	980	180			

BC-fed, piglets separated from the sow and fed bovine colostrum; MR-fed, piglets separated from the sow and fed a commercial milk replacer; Sow-Milk, piglets kept with the sow; Treat, treatment.

### Gene expression levels in the intestinal mucosa

**Fig. 3:**
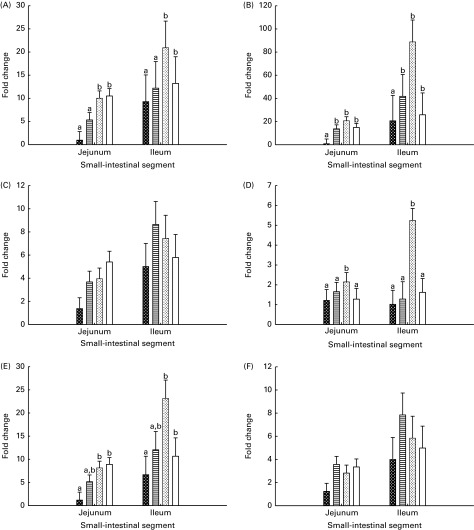
Gene expression levels of Toll-like receptor-4 (A), cyclo-oxygenase-2 (B), *TNF-α* (C), *IL-2* (D), *IL-10* (E) and transforming growth factor-β1 (F) in the intestinal mucosa of piglets. Values are least-squares means with their standard errors represented by vertical bars. ^a,b^Mean values with unlike letters were significantly different (*P*≤ 0·05). Four piglets were analysed per treatment and small-intestinal segment. Base (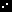), piglets killed at the beginning of the experiment; BC-fed (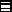), piglets separated from the sow and fed bovine colostrum; MR-fed (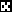), piglets separated from the sow and fed a commercial milk replacer; Sow-Milk (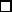), piglets kept with the sow. (A) *P*_treat_= 0·011; *P*_seg_= 0·010; *P*_treat × seg_= 0·726. (B) *P*_treat_= 0·046; *P*_seg_= 0·007; *P*_treat × seg_= 0·183. (C) *P*_treat_= 0·100; *P*_seg_= 0·009; *P*_treat × seg_= 0·500. (D) *P*_treat_= 0·009; *P*_seg_= 0·171; *P*_treat × seg_= 0·074. (E) *P*_treat_= 0·022; *P*_seg_= 0·005; *P*_treat × seg_= 0·191. (F) *P*_treat_= 0·161; *P*_seg_= 0·002; *P*_treat × seg_= 0·720.

Gene expression levels in the jejunal and ileal mucosa of piglets are shown in Fig. 3. The genes were expressed at higher (*P*_seg_≤ 0·010) levels in the ileal mucosa than in the jejunal mucosa, except for the *IL-2* gene. The expression level of *TLR-4* was lower (*P*≤ 0·037) in the jejunal and ileal mucosa of Base and BC-fed piglets than in those of MR-fed and Sow-Milk piglets. The expression level of *IL-2* was higher (*P*≤ 0·051) in the intestinal mucosa of piglets from the MR-fed group than in that of piglets from the other treatment groups. Compared with that in piglets from the other treatment groups, the expression level of cyclo-oxygenase-2 (*COX-2*) was lower (*P*≤ 0·047) in the intestinal mucosa of Base piglets and was significantly higher in MR-fed piglets. The level of *IL-10* expression was lower (*P*≤ 0·036) in the intestinal mucosa of Base piglets than in that of MR-fed and Sow-Milk piglets, but did not differ from that in BC-fed piglets. No significant effect of treatment was observed on the expression levels of *TNF-α* and transforming growth factor-β1 (*TGF-β1*).

Data on gene expression levels in the jejunal and ileal mucosa samples collected from the PIOC model were found to be invalid in the present study due to the inconsistent gene expression patterns measured. Compared with those in the control, gene expression levels in the mucosa of ETEC F18-inoculated segments were higher, lower or similar, with no clear patterns. Therefore, the values are not reported or discussed here.

## Discussion

About 3 weeks of age, piglets have the lowest concentrations of maternal antibodies acquired from the sow milk and their immune system is not as well developed as that of adults^(^ ^7^ ^)^. Thus, due to similarities, the use of 23-d-old piglets in the present study could provide some information regarding weak piglets having an incompetent intestinal immune system. Indeed, at this age, piglets are also highly susceptible to ETEC F18, given that F18 receptors on porcine enterocytes are functionally active (F18 receptors are not yet fully active in piglets under about 3 weeks of age)^(^ ^19^ ^)^. Several studies have reported positive effects of BC-supplemented diets on the immune responses of piglets^(^ ^12^ ^,^ ^20^ ^)^. In general, BC treatment was mainly used to provide additional Ig to weaned piglets following the loss of passive protection provided by the sow milk. However, data on the efficacy of BC supplements in enhancing antibody responses in piglets after weaning have been inconsistent. Boudry *et al.* ^(^ ^12^ ^)^ reported that oral supplementation with BC (piglets received 5 g of BC orally daily for 3 weeks) increased serum IgA concentrations (after 3 weeks of BC supplementation, but not before), but transiently decreased total IgG concentrations and had no influence on total IgM concentrations. In addition, total IgG, IgA and IgM concentrations in intestinal fluid samples were not affected by BC supplementation. In another study, Boudry *et al.* ^(^ ^20^ ^)^ demonstrated that BC whey supplementation (20 g/kg of BC whey incorporated in the diet during the first 2 weeks after weaning and 10 g/kg for the next 2 weeks) increased circulating IgA concentrations by 25 % (during the 1st week after weaning, but not during the remaining experimental period) and had no impact on total IgG and IgM concentrations. Taking this into consideration, feeding a commercial BC preparation as the only dietary ingredient, rather than as a supplement, could be speculated to have beneficial effects on the immune responses of artificially reared weak piglets before weaning to a greater extent. It is known that the nutritional composition of diet, particularly protein, affects the immunocompetence of animals^(^ ^21^ ^)^. There was a substantial difference in the protein content of BC and that of the other two diets in the present study. Indeed, protein in BC is mainly composed of Ig (70–80 % of the total protein content)^(^ ^4^ ^)^ and hence any influence of BC on the immune system of piglets can reasonably be assumed to be due to Ig (and other immune factors such as cytokines) in BC.

In the present study, feeding a commercial BC product rather than a MR was found to have the capacity to reduce the frequency of diarrhoea in piglets and no differences were actually found in the frequency of diarrhoea in BC-fed piglets and Sow-Milk piglets. Corresponding results have been reported by Huguet *et al.* ^(^ ^22^ ^)^, who found that feeding a BC-supplemented diet reduced diarrhoeal episodes in weaned piglets.

BC has been reported to contain growth factors essential for promoting the growth and development of newborn piglets^(^ ^4^ ^)^. Yet, feeding BC could not improve the growth performance of piglets in the present study, as evidenced by the finding that piglets staying with their dams gained more weight than those artificially reared on BC.

All piglets used in the present study were genetically susceptible to ETEC expressing F18 fimbriae. Previous studies have shown that BC exhibits antimicrobial activity through its functional components such as Ig^(^ ^4^ ^,^ ^13^ ^)^. In accordance with this, bacterial counts in the faecal and digesta samples revealed that feeding BC resulted in lower counts of haemolytic *E. coli* (indicator of ETEC) compared with feeding a commercial MR. The mean haemolytic *E. coli* count in the faeces was 5·97 (se 0·72) log cfu/g in BC-fed piglets, 6·58 (se 1·34) log cfu/g in MR-fed piglets and 5·87 (se 0·42) log cfu/g in Sow-Milk piglets. The mean haemolytic *E. coli* count in the digesta across the GIT was 6·34 (se 1·63) log cfu/g in BC-fed piglets, 7·05 (se 1·90) log/cfu in MR-fed piglets and 5·28 (se 0·78) log/cfu in Sow-Milk piglets. These values are within the normal range for haemolytic *E. coli* enumerated in the faeces and intestinal content of piglets after weaning^(^ ^23^ ^,^ ^24^ ^)^. Consistent with the bacterial counts in the faecal and GIT digesta samples, jejunal and ileal tissue samples collected from BC-fed (and Sow-Milk) piglets exhibited a significant reduction in the recovery of *E. coli* from the tissue when compared with samples collected from MR-fed piglets. Data from the PIOC experiment also showed that, relatively to feeding a MR, feeding BC is associated with a lower colonisation of *E. coli* in the intestinal tissue after inoculation with ETEC F18. This is in agreement with the results of earlier studies reporting an inhibitory effect of BC on the adherence of enterohaemorrhagic *E.* *coli* and enteropathogenic *E. coli* to the intestinal mucosa of mice and HEp-2 cells, respectively^(^ ^25^ ^,^ ^26^ ^)^. In another *in vitro* study, pre-treatment with BC was found to decrease the attachment of Shiga toxin-producing *E. coli* O111 to colon-derived cell lines (HT-29)^(^ ^27^ ^)^. Altogether, we infer that BC is capable of inhibiting the attachment and/or colonisation of ETEC in the intestinal mucosa of piglets.

It has previously been shown that BC feeding could preserve the balanced microbiota in the intestine of piglets^(^ ^4^ ^,^ ^28^ ^)^. Accordingly, the results of the present study revealed that the LAB:haemolytic *E. coli* and LAB:coliform bacteria ratios in the faecal and digesta samples collected from BC-fed piglets were higher than those in samples collected from MR-fed piglets. This further provides an indication that BC is capable of inhibiting the growth of coliform bacteria and ETEC in the gut of piglets. The results also revealed that the LAB:haemolytic *E. coli* ratio in the faeces decreased with time after experimental treatment. However, the decrease was mainly observed in piglets that were separated from the sows, suggesting that separation from the sows altered the balanced gut microbiota in piglets. As has been observed in the present study, previous studies have reported that the number of enteric bacterial pathogens increase, whereas LAB population levels are suppressed after weaning^(^ ^4^ ^,^ ^10^ ^,^ ^29^ ^)^.

As has been discussed above, BC is an important source of Ig that may provide the host with passive immunity against infectious pathogens^(^ ^4^ ^,^ ^13^ ^)^. In the present study, the concentrations of Ig in the BC were much higher than those in the sow milk and MR. Yet, the resulting concentrations of Ig in the mucosa of BC-fed piglets did not differ significantly from those in the mucosa of MR-fed or Sow-Milk piglets. Similar results were reported by Boudry *et al.* ^(^ ^12^ ^)^, as has been mentioned above. The inability of porcine Ig receptors on the surface of enterocytes to efficiently bind to the bovine Ig^(^ ^30^ ^)^ may explain this finding. The bovine colostral Ig that are not absorbed by the intestine (remaining in the intestinal lumen) may, however, still provide passive local immunity to piglets by binding to the antigens and protecting the intestinal tissue through immune exclusion^(^ ^13^ ^)^. In the present study, feeding BC to the piglets did not result in differences in terms of circulatory Ig concentrations. The low capacity of the intestine to absorb bovine Ig^(^ ^30^ ^)^ and/or gut closure^(^ ^12^ ^,^ ^31^ ^)^ may explain the poor transfer of bovine Ig from the gut to the bloodstream in piglets during the period after separation from the sows. Irrespective of the treatment, the concentration of plasma IgG was higher on day 1 than on day 8 of the experiment. The decrease in total plasma IgG concentrations could be associated with the age effect or decay of passively acquired maternal IgG^(^ ^12^ ^)^. In accordance with the systemic Ig, the concentrations of total IgA were higher in the jejunal and ileal mucosa of piglets from the Base group, killed at the beginning of the experiment, than in that of piglets from the other treatment groups, which were killed 8 d later.

Changes in the diet and environment of piglets after separation from the sows have been known to be associated with an increased number of pathogenic bacteria in the various regions of the GIT^(^ ^10^ ^)^. Through TLR, these bacteria are able to produce signals that up-regulate the expression of pro-inflammatory factors and stimulate host immune responses^(^ ^32^ ^)^. In the present study, BC was able to reduce the intestinal colonisation by ETEC and, accordingly, the increased expression of *TLR-4* was not observed in the intestinal mucosa of BC-fed piglets. The release of cytokines or other inflammatory factors upon recognition of Gram-negative pathogenic bacteria is one of the most important effects of TLR-4 activation^(^ ^32^ ^)^. In the case of BC-fed piglets, a low activation of TLR-4 by ETEC may therefore be implicated in the lower expression of pro-inflammatory mediators, apparently as in the lower expression levels of *IL-2* (inflammatory mediators in response to bacterial infections^(^ ^33^ ^)^) in the intestinal mucosa. TNF-α is an inflammatory cytokine that is normally up-regulated upon antigen stimulation^(^ ^27^ ^)^. Unlike those of *IL-2*, the expression levels of *TNF-α* in the intestinal mucosa did not differ among the treatment groups in the present study. BC is known to contain several potential pro-inflammatory components, including TNF-α and IL-6^(^ ^4^ ^)^. Therefore, pre-treatment with BC increases the production of TNF-α and IL-6 in both antigen-stimulated and unstimulated human monocytic THP-1 cells (ATCCIIB-202)^(^ ^27^ ^)^. Many immunomodulatory factors such as TNF-α and IL-10 are also present in the sow milk^(^ ^5^ ^)^. Taken together, the potential of BC and sow milk to up-regulate the expression of *TNF-α* may therefore explain the lack of difference in the expression levels of this cytokine among the treatment groups, although the small intestine of BC-fed and Sow-Milk piglets was less colonised by the *E. coli* compared with that of MR-fed piglets. IL-10 is an anti-inflammatory cytokine that plays a critical immunoregulatory role (balances immune activation) during immune responses. There are diverse stimuli that can induce the production of this cytokine, among which are bacteria and bacterial products (enterotoxins)^(^ ^34^ ^)^. Concomitant with that of *IL-10*, the expression of *TGF-β1*, which is an essential signal for the generation of regulatory T cells and T-helper 17 cells, is induced by bacterial infections^(^ ^35^ ^)^. In conjunction with the higher population levels of *E. coli* in the intestine of MR-fed piglets that might lead to higher expression levels of *IL-10* and *TGF-β1* in the mucosa, the presence of IL-10^(^ ^5^ ^)^ and TGF-β1^(^ ^4^ ^,^ ^5^ ^)^ in the BC and sow milk appeared to be responsible for the increased expression levels of these immune mediators in the intestinal mucosa of BC- and sow milk-fed piglets^(^ ^36^ ^)^. This condition may explain the insignificant differences in *IL-10* and *TGF-β1*expression levels among the treatment groups in the present study.

An interesting finding from the present study is that genes (*TLR-4*, *COX-2*, *TNF-α*, *IL-10* and *TGF-β1*) were expressed at higher levels in the ileal mucosa than in the jejunal mucosa of piglets, irrespective of the treatment. Consistent with this, the concentrations of total IgA and IgM were higher in the ileal mucosa than in the jejunal mucosa. Based on data from the PIOC experiment, in which more *E. coli* were enumerated in ileal tissue than in jejunal tissue, we infer that the immune responses may partly be affected by the degree of colonisation of bacterial pathogens in the gut. This is in accordance with the results of studies of Wang *et al.* ^(^ ^35^ ^)^ and Daudelin *et al.* ^(^ ^37^ ^)^, who reported that the colonisation of *E. coli* or other bacterial pathogens in the intestine is responsible for the stimulation of the host-related immune responses.

In conclusion, feeding BC to the piglets reduced the intestinal colonisation of ETEC and modulated the intestinal mucosal immune system when compared with feeding a MR. Rearing piglets on BC was as good as conventional rearing by sows in terms of optimisation of the immune defence of weak piglets. Hence, future research should consider the potential of BC supplementation in weak piglets.
